# Infused autograft lymphocyte-to-monocyte ratio and survival in T-cell lymphoma post-autologous peripheral blood hematopoietic stem cell transplantation

**DOI:** 10.1186/s13045-015-0178-5

**Published:** 2015-07-03

**Authors:** Luis F. Porrata, David J. Inwards, Stephen M. Ansell, Ivana N. Micallef, Patrick B. Johnston, William J. Hogan, Svetomir N. Markovic

**Affiliations:** Division of Hematology, Department of Medicine, Mayo Clinic, 200 first St. SW, Rochester, MN 55905 USA

**Keywords:** Autograft absolute lymphocyte-to-monocyte count ratio, Survival, Autologous peripheral hematopoietic stem cell transplantation, T-cell lymphomas

## Abstract

**Background:**

The infused autograft lymphocyte-to-monocyte ratio (A-LMR) is a prognostic factor for survival in B-cell lymphomas post-autologous peripheral hematopoietic stem cell transplantation (APHSCT). Thus, we set out to investigate if the A-LMR is also a prognostic factor for survival post-APHSCT in T-cell lymphomas.

**Methods:**

From 1998 to 2014, 109 T-cell lymphoma patients that underwent APHSCT were studied. Receiver operating characteristic (ROC) and area under the curve (AUC) were used to identify the optimal cut-off value of A-LMR for survival analysis and *k*-fold cross-validation model to validate the A-LMR cut-off value. Univariate and multivariate Cox proportional hazard models were used to assess the prognostic discriminator power of A-LMR.

**Results:**

ROC and AUC identified an A-LMR ≥ 1 as the best cut-off value and was validated by *k*-fold cross-validation. Multivariate analysis showed A-LMR to be an independent prognostic factor for overall survival (OS) and progression-free survival (PFS). Patients with an A-LMR ≥ 1.0 experienced a superior OS and PFS versus patients with an A-LMR < 1.0 [median OS was not reached vs 17.9 months, 5-year OS rates of 87 % (95 % confidence interval (CI), 75–94 %) vs 26 % (95 % CI, 13–42 %), *p* < 0.0001; median PFS was not reached vs 11.9 months, 5-year PFS rates of 72 % (95 % CI, 58–83 %) vs 16 % (95 % CI, 6–32 %), *p* < 0.0001].

**Conclusions:**

A-LMR is also a prognostic factor for clinical outcomes in patients with T-cell lymphomas undergoing APHSCT.

## Introduction

The absolute lymphocyte count (ALC), as a surrogate marker of host immunity, and the absolute monocyte count (AMC), as a surrogate marker of tumor microenvironment, have been reported to be prognostic factors in B-cell lymphomas [1–5]. Similarly, recent studies have shown that both the ALC and AMC have the prognostic ability to predict clinical outcomes in T-cell lymphomas [6–8]. In autologous peripheral hematopoietic stem cell transplantation (APHSCT), day 15 absolute lymphocyte count (ALC-15) recovery has been associated with improved survival post-transplant not only in B-cell lymphomas [9, 10] but also in T-cell lymphomas [11]. ALC-15 recovery directly depends on the amount of autograft absolute lymphocyte count (A-ALC) collected during stem cell collection and infused in conjunction with stem cells [12–14]. Furthermore, the day-15 absolute monocyte count (AMC-15) is also related to the amount of autograft absolute monocyte count (A-AMC) collected and infused to patients undergoing APHSCT [15]. Both ALC-15 and AMC-15 have been reported to affect survival post-APHSCT [15]. We combined the A-ALC and the A-AMC as a simple biomarker integrating the host immunity (i.e., A-ALC) and tumor microenvironment (i.e., A-AMC) into the autograft lymphocyte-to-monocyte ratio (A-LMR) and reported that A-LMR directly affects clinical outcomes in both diffuse large B-cell lymphoma [16] and classical Hodgkin lymphoma [17] patients undergoing APHSCT. Thus, we set out to investigate if the A-LMR also affects survival in T-cell lymphoma patients undergoing APHSCT.

## Results

### Patients’ characteristics

The median age at the time of transplant for this cohort of 109 T-cell lymphoma patients was 56 years (range: 19–77 years). The distribution of additional baseline characteristics for these patients is presented in Table 1. The median follow-up for the entire cohort was 21.5 months (range: 1–147.8 months) and for the living patients (*N* = 70) was 39.6 months (range: 1.8–147.8 months). Fifty-eight (53 %) of the patients received their APHSCT up front after finishing induction chemotherapy. Two patients received SMILE (methotrexate, leucovorin, ifosfamide, dexamethasone, etoposide, and pegaspargase) chemotherapy and the rest CHOP (cyclophosphamide, doxorubicin, vincristine, and prednisone) chemotherapy as their induction chemotherapy. The salvage chemotherapy included CDE, DHAP, and ICE. The day 100 transplant-related mortality (TRM) for the cohort of patients was 1.8 % (2/109). Thirty-one patients died due to relapse/progression of lymphoma. Six patients died of causes unrelated to lymphoma, excluding the two patients that died in the first 100 days post-APHSCT.

**Table 1:** Baseline characteristics in the training set and validation set

Variable	A-LMR ≥ 1 (*N* = 59)	A-LMR < 1 (*N* = 50)	*p* value
At diagnosis			
Age, years, median (range)	53 (18–71)	56 (20–75)	0.2
Gender			0.2
Male	38 (64 %)	25 (50 %)	
Female	21 (36 %)	25 (50 %)	
LDH (U/L), median (range)	211 (116–928)	276.5 (137–1539)	0.04
Extra-nodal site			0.05
0	7 (12 %)	6 (12 %)	
1	49 (83 %)	34 (68 %)	
2	3 (5 %)	10 (20 %)	
B symptoms			0.9
Yes	17 (29 %)	15 (30 %)	
No	42 (71 %)	35 (70 %)	
Stage			<0.001
I	2 (3 %)	1 (2 %)	
II	26 (44 %)	5 (10 %)	
III	9 (15 %)	12 (24 %)	
IV	22 (38 %)	32 (64 %)	
Bone marrow involvement			0.5
Yes	15 (25 %)	16 (32 %)	
No	44 (75 %)	34 (68 %)	
Bulky disease			0.8
Yes	3 (5 %)	6 (6 %)	
No	56 (95 %)	47 (94 %)	
Hemoglobin (g/dl), median (range)	12.5 (8.1–13.1)	12.5 (6.6–16.9)	0.6
Platelets × 10^9^/l, median (range)	231 (14–626)	214 (31–719)	0.06
IPI score			0.01
0	13 (22 %)	2 (4 %)	
1	19 (32 %)	12 (24 %)	
2	17 (29 %)	18 (36 %)	
3	8 (13 %)	10 (20 %)	
4	1 (2 %)	7 (14 %)	
5	1 (2 %)	1 (2 %)	
Performance status			0.3
0	7 (12 %)	4 (8 %)	
1	45 (76 %)	43 (86 %)	
2	5 (8 %)	3 (6 %)	
3	2 (45 %)	0 (0 %)	
T-cell lymphoma histologies			0.9
Anaplastic large cell	10 (17 %)	5 (13 %)	
Angioimmunoblastic	15 (25 %)	15 (27 %)	
Enteropathy-associated	6 (10 %)	3 (6 %)	
Hepatosplenic	1 (2 %)	1 (2 %)	
NK/T	7 (12 %)	6 (12 %)	
Panniculitis	4 (7 %)	3 (6 %)	
Peripheral	16 (27 %)	17 (34 %)	
IPI factors			
Age, years			0.3
60	18 (31 %)	20 (40 %)	
≤60	41 (69 %)	30 (60 %)	
LDH (U/L)			0.03
Normal	34 (58 %)	18 (36 %)	
Abnormal	25 (42 %)	32 (64 %)	
Performance status			0.3
1	7 (12 %)	3 (6 %)	
≤1	52 (88 %)	47 (94 %)	
Extra-nodal disease			0.02
1	3 (5 %)	10 (20 %)	
≤1	56 (95 %)	40 (80 %)	
Stage			<0.001
I/II	28 (47 %)	6 (12 %)	
III/IV	31 (53 %)	44 (88 %)	
IPI index			0.03
2	10 (17 %)	18 (36 %)	
≤2	49 (83 %)	32 (64 %)	
Initial chemotherapy			0.9
CHOP	58 (98 %)	49 (98 %)	
SMILE	1 (2 %)	1 (2 %)	
Salvage chemotherapy (*N* = 51)			0.2
CDE	2 (9 %)	0 (0 %)	
DHAP	5 (21 %)	8 (29 %)	
ICE	16 (70 %)	20 (71 %)	
Up-front transplant			0.09
Yes	36 (61 %)	22 (44 %)	
No	23 (39 %)	28 (56 %)	
At transplant			
Pre-transplant clinical status			<0.001
CR	51 (86 %)	25 (50 %)	
PR	8 (15 %)	25 (50 %)	
Plerixafor			0.8
Yes	21 (36 %)	19 (38 %)	
No	38 (64 %)	31 (62 %)	
Infused CD34, median (range)	5.42 (2.25–13.44)	5.12 (2.04–15.65)	0.9
Number of collections			0.08
1	7 (12 %)	12 (24 %)	
2	19 (32 %)	15 (30 %)	
3	19 (32 %)	9 (18 %)	
4	7 (12 %)	11 (22 %)	
5	1 (2 %)	2 (4 %)	
6	3 (5 %)	0 (0 %)	
7	2 (3 %)	0 (0 %)	
8	1 (2 %)	1 (2 %)	
A-ALC, median (range)	0.68 (0.21–3.83)	0.44 (0.06–1.40)	<0.001
A-AMC, median (range)	0.45 (0.10–1.42)	0.78 (0.20–1.60)	<0.001
ALC-15, median (range)	0.71 (0.10–2.31)	0.41 (0.10–1.18)	<0.001
AMC-15, median (range)	0.43 (0.04–1.19)	0.90 (0.20–2.31)	<0.001
LMR-15	1.71 (0.24–15.5)	0.57 (0.08–3.18)	<0.001

Abbreviations: *A-ALC* autograft absolute lymphocyte count; *ALC-15* day 15 absolute lymphocyte count post-autologous peripheral hematopoietic stem cell transplantation; *A-AMC* autograft absolute monocyte count; *AMC-15* day 15 absolute monocyte count post-autologous peripheral hematopoietic stem cell transplantation; *A-LMR* autograft lymphocyte/monocyte ratio; *CDE* cyclophosphamide, doxorubicin, and etoposide; *CR* complete response; *CHOP* cyclophosphamide, doxorubicin, vincristine, and prednisone; *DHAP* dexamethasone, cytarabine, and cisplatin; *ICE* ifosfamide, carboplatin, and etoposide; *IPI* International Prognostic Index; *LDH* lactate dehydrogenase; *LMR- 15* day 15 lymphocyte/monocyte ratio post-autologous peripheral hematopoietic stem cell transplantation; *PR* partial response; *SMILE* methotrexate, leucovorin, ifosfamide, dexamethasone, etoposide, and pegaspargase

### Cut-off values for A-ALC, A-AMC, A-LMR, ALC-15, AMC-15, and LMR-15 for survival analysis

**Fig. 1:**
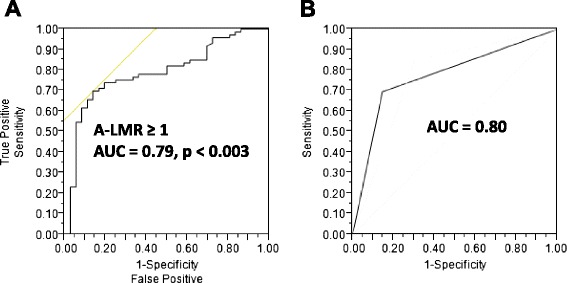
**a** Receiver operating characteristics (ROC) curves and area under the curve (AUC) for autograft lymphocyte-to-monocyte ratio (A-LMR). **b** *k*-fold cross-validation ROC and AUC for A-LMR in the training set

**Fig. 2:**
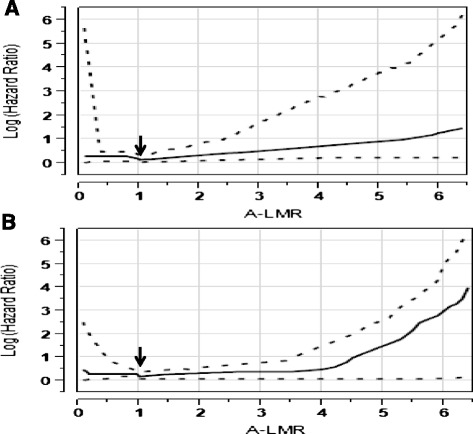
**a** Determination of hazard ratio associated with different levels of autograft lymphocyte-to-monocyte ratio (A-LMR) as a continuous variable for overall survival. **b** Determination of hazard ratio associated with different levels of A-LMR as continuous variable for progression-free survival

Receiver operating characteristic (ROC) curves and area under the curve (AUC) were used to determine the optimal cut-off points for A-ALC, A-AMC, A-LMR, ALC-15, AMC-15, and day 15 lymphocyte/monocyte ratio post-autologous peripheral hematopoietic stem cell transplantation (LMR-15) based on their utility as a marker for the clinical binary outcome of death/survival. The A-LMR ≥ 1 had an AUC of 0.79 (95 % confidence interval (CI), 0.73–0.85) with a sensitivity of 70 % (95 % CI, 66–76 %) and specificity of 87 % (95 % CI, 83–91 %), *p* < 0.003 (Fig. 1a). The A-ALC ≥ 0.5 × 10^9^ cells/kg had an AUC of 0.69 (95 % CI, 0.61–0.77) with a sensitivity of 78 % (95 % CI, 70–86 %) and specificity of 68 % (95 % CI, 59–77 %), *p* < 0.04. The A-AMC < 0.6 × 10^9^ cells/kg had an AUC of 0.71 (95 % CI, 0.62–0.80) with a sensitivity of 64 % (95 % CI, 55–75 %) and specificity of 75 % (95 % CI, 67–83 %), *p* < 0.0006. The ALC-15 ≥ 500 cells/μl had an AUC of 0.78 (95 % CI, 0.70–0.86) with a sensitivity of 77 % (95 % CI, 69–85 %) and specificity of 78 % (95 % CI, 70–86 %), *p* < 0.0001. The AMC-15 < 500 cells/μl had an AUC of 0.66 (95 % CI, 0.59–0.73) with a sensitivity of 63 % (95 % CI, 54–72 %) and specificity of 84 % (95 % CI, 77–91 %), *p* < 0.01. The LMR-15 ≥ 1 had an AUC of 0.82 (95 % CI, 0.73–0.89) with a sensitivity of 75 % (95 % CI, 67–83 %) and specificity of 81 % (95 % CI, 74–88 %), *p* < 0.0001. An internal validation of A-LMR, A-ALC, A-AMC, ALC-15, AMC-15, and LMR-15 performances as markers for the clinical binary outcomes of death/survival was performed using *k*-fold cross-validation with *k* = 10. We obtained an average AUC of 0.80 (95 %CI, 0.73–0.88) over the ten validation sets for A-LMR with a standard deviation of ±0.02. We report the ROC for the complete dataset used in the tenfold procedure, by collecting the A-LMR obtained on each fold. For A-LMR, the cross-validation ROC (Fig. 1b) showed an AUC of 0.80 (95 % CI, 0.68–0.92). The similar areas under the curves from the empirical ROC and the cross-validation ROC, as well as for both overall survival (OS) (Fig. 2a) and progression-free survival (PFS) (Fig. 2b) an A-LMR ≥ 1 was the point of change for the hazard ratio from favorable to unfavorable, support the use of A-LMR ≥ 1 as the cut-off value as marker of the binary clinical outcome of death/survival. In similar fashion, we obtained an average AUC for A-ALC of 0.67 (95 % CI, 0.59–0.75), A-AMC of 0.68 (95 % CI, 0.60–0.76), ALC-15 of 0.76 (95 % CI, 0.68–0.84), AMC-15 of 0.67 (95 % CI, 0.60–0.74), and LMR-15 of 0.83 (95 % CI, 0.76–0.90). We report the ROC for the complete dataset used in the tenfold procedure, by collecting the A-ALC, A-AMC, ALC-15, AMC-15, and LMR-15 obtained on each fold. For A-ALC, the cross validation ROC showed an AUC of 0.68 (95 %CI, 0.61–0.75), for A-AMC of 0.69 (95 % CI, 0.61–0.78), for ALC-15 of 0.77 (95 % CI, 0.69–0.85), for AMC-15 of 0.68 (95 % CI, 0.61–0.75), and for LMR-15 of 0.84 (95 % CI, 0.77–0.91). The close values of AUC from the empirical ROC and the cross-validation ROC support the use of A-ALC ≥ 0.5 × 10^9^ cells/kg, A-AMC < 0.6 × 10^9^ cells/kg, ALC-15 ≥ 500 cells/μl, AMC-15 < 500 cells/μl, and LMR-15 ≥ 1 as the cut-off values to test the binary clinical outcome of death/survival.

### Correlation between A-ALC and ALC-15, A-AMC and AMC-15, and A-LMR and LMR-15

**Fig. 3:**
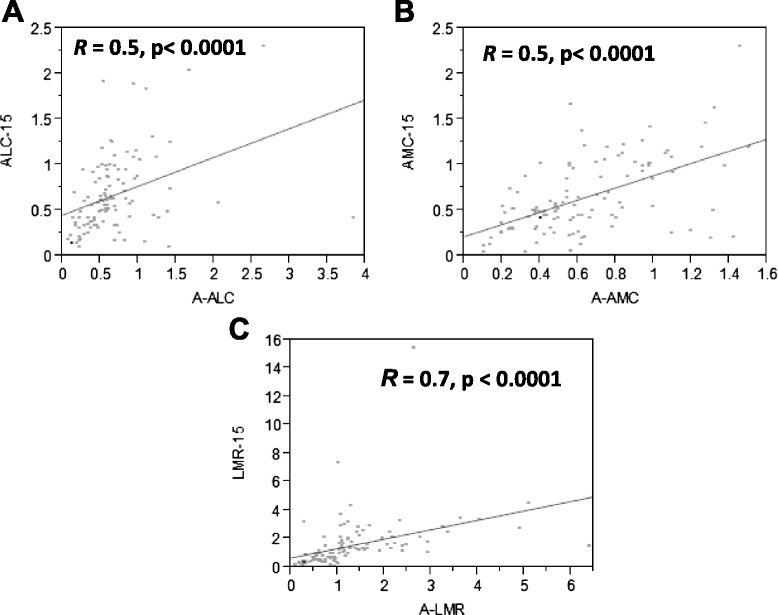
Scatter plots for: **a** autograft absolute lymphocyte count (A-ALC) and day 15 absolute lymphocyte count (ALC-15), **b** autograft absolute monocyte count (A-AMC) and day 15 absolute monocyte count (AMC-15), and **c** autograft absolute lymphocyte/monocyte ration (A-LMR) and day 15 absolute lymphocyte/monocyte ratio (LMR-15)

In B-cell lymphomas undergoing APHSCT, we reported a positive correlation between A-ALC and ALC-15, A-AMC and AMC-15, and A-LMR and LMR-15. In this cohort of T-cell lymphoma patients, a strong positive correlation was also observed between A-ALC and ALC-15 (*R* = 0.5, *p* < 0.0001) (Fig. 3a), between A-AMC and AMC-15 (*R* = 0.5, *p* < 0.0001) (Fig. 3b), and between A-LMR and LMR-15 (*R* = 0.7, *p* < 0.0001) (Fig. 3c). No correlation was identified between the infused CD34 and ALC-15 (*R* = 0.01, *p* = 0.9), infused CD34 and AMC-15 (*R* = 0.13, *p* = 0.2), or infused CD34 and LMR-15 (*R* = 0.10, *p* = 0.3).

### Predictors for OS and PFS

Using the univariate Cox regression analysis, the following variables were predictors for OS: B symptoms, platelets, extra-nodal disease, lactate dehydrogenase (LDH), stage, International Prognostic Index (IPI), complete response (CR) prior to APHSCT, A-ALC, A-AMC, A-LMR, ALC-15, AMC-15, and LMR-15 (Table 2). For PFS, the following variables were predictors: hemoglobin, platelets, extra-nodal disease, LDH, stage, IPI, CR prior to APHSCT, A-ALC, A-AMC, A-LMR, ALC-15, AMC-15, and LMR-15 (Table 2). Since extra-nodal disease, LDH, and stage are components of IPI, IPI was only included in the multivariate analysis. Multivariate analysis identified the following predictors for OS and PFS: A-ALC, A-LMR, A-AMC, and CR prior to APHSCT (Table 3). To avoid the problem of colinearity due to the strong positive correlation between A-ALC and ALC-15, A-AMC and AMC-15, and A-LMR and LMR-15, we substituted ALC-15 for A-ALC, AMC-15 for A-AMC, and LMR-15 for A-LMR and tested them against the other predictors (CR prior to APHSCT, extra-nodal disease, hemoglobin, IPI, LDH, platelets, and stage) in the multivariate analysis. ALC-15, AMC-15, and LMR-15 remained independent predictors for OS and PFS: for OS [ALC-15: hazard ratio (HR) of 0.256, 95 % CI, 0.094–0.665, *p* < 0.005; AMC-15: HR of 0.351, 95 % CI, 0.169–0.820, *p* < 0.01; and LMR-15: HR of 0.307, 95 % CI, 0.102–0.820, *p* < 0.02] and for PFS [ALC-15: HR of 0.439, 95 % CI, 0.200–0.967, *p* < 0.04; AMC-15: HR of 0.383, 95 % CI, 0.175–0.867, *p* < 0.01; and LMR-15: HR of 0.408, 95 % CI, 0.200–0.823, *p* < 0.01].

**Table 2:** Univariate analysis for overall survival (OS) and progression-free survival (PFS)

Variable	OS			PFS		
	HR	95 % CI	*p*	HR	95 % CI	*p*
Age > 60 years	1.532	0.784–2.900	0.2	1.400	0.789–2.422	0.2
Female versus male	1.153	0.606–2.170	0.7	1.359	0.787–2.342	0.3
B symptoms	1.919	1.094–3.615	0.05	1.425	0.790–2.485	0.2
Bulky disease	1.007	0.164–3.294	0.9	1.109	0.270–3.024	0.9
Hemoglobin < 12.0 g/dl	1.636	0.862–3.072	0.1	1.858	1.076–3.193	0.03
Platelet < 150 × 10^9^/l	2.566	1.312–4.862	0.007	2.890	1.671–5.047	<0.001
Extra-nodal sites > 1	3.043	1.354–6.197	0.009	2.332	1.104–4.470	0.03
LDH (U/L)	6.695	3.001–17.77	<0.001	4.510	2.440–8.989	<0.001
Performance status > 1	1.788	0.674–3.972	0.2	2.033	0.885–4.089	0.09
Stage III/IV	3.817	1.630–11.160	<0.001	2.571	1.393–5.431	0.004
IPI > 2	4.442	2.342–8.459	<0.001	3.734	2.146–6.444	<0.001
Positive bone marrow	1.670	0.856–3.153	0.1	1.294	0.709–2.271	0.4
Infused CD34	0.893	0.758–1.032	0.1	0.950	0.832–1.070	0.4
CR prior to APHSCT	0.164	0.081–0.314	<0.001	0.186	0.106–0.321	<0.001
A-ALC ≥ 0.5 × 10^9^/kg	0.358	0.188–0.676	0.002	0.294	0.167–0.511	<0.001
A-AMC ≥ 0.6 × 10^9^/kg	3.207	1.585–7.175	<0.001	2.254	1.285–4.116	0.004
A-LMR ≥ 1	0.155	0.066–0.322	<0.001	0.196	0.104–0.353	<0.001
ALC-15 ≥ 0.5 cells/μl	0.158	0.072–0.304	<0.001	0.246	0.139–0.430	<0.001
AMC-15 ≥ 0.5 cells/μl	3.909	1.881–9.160	<0.001	2.645	1.489–4.918	<0.001
LMR-15	0.143	0.061–0.497	<0.001	0.214	0.116–0.378	<0.001
Plerixafor	0.954	0.497–1.925	0.7	0.886	0.510–1.587	0.8

Abbreviations: *A-ALC* autograft absolute lymphocyte count, *ALC-15* day 15 absolute lymphocyte count post-autologous peripheral hematopoietic stem cell transplantation, *A-AMC* autograft absolute monocyte count, *AMC-15* day 15 absolute monocyte count post-autologous peripheral hematopoietic stem cell transplantation, *A-LMR* autograft lymphocyte/monocyte ratio, *LMR-15* day 15 lymphocyte/monocyte ratio post-autologous peripheral hematopoietic stem cell transplantation, *CR* complete response, *IPI* International Prognostic Index, *LDH* lactate dehydrogenase

**Table 3:** Multivariate analysis for overall survival (OS) and progression-free survival (PFS)

Variable	OS			PFS		
	HR	95 % CI	*p*	HR	95 % CI	*p*
A-ALC ≥ 0.5 × 10^9^/kg	0.401	0.231–0.972	0.04	0.439	0.271–0.902	0.03
A-AMC < 0.6 × 10^9^/kg	0.502	0.100–0.985	0.04	0.490	0.288–0.911	0.03
A-LMR ≥ 1	0.258	0.105–0.561	<0.001	0.298	0.112–0.482	<0.001
CR prior to APHSCT	0.356	0.166–0.731	0.005	0.401	0.211–0.681	0.008
Hemoglobin < 12 g/dl				1.592	0.861–2.935	0.1
IPI > 2	2.377	1.163–4.972	0.02	1.398	0.705–2.771	0.3
Platelet < 150 × 10^9^/l	1.297	0.627–2.607	0.5	1.164	0.590–2.268	0.7

Abbreviations: *A-ALC* autograft absolute lymphocyte count, *A-AMC* autograft absolute monocyte count, *A-LMR* autograft lymphocyte/monocyte ratio, *A-LMR* autograft lymphocyte/monocyte ratio, *CR* complete response, *IPI* International Prognostic Index, *LDH* lactate dehydrogenase

### Survival outcomes based on A-LMR

**Fig. 4:**
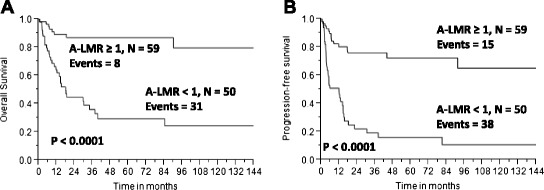
**a** Overall survival based on autograft lymphocyte-to-monocyte ratio (A-LMR). **b** Progression-free survival based on autograft lymphocyte-to-monocyte ratio (A-LMR)

**Fig. 5:**
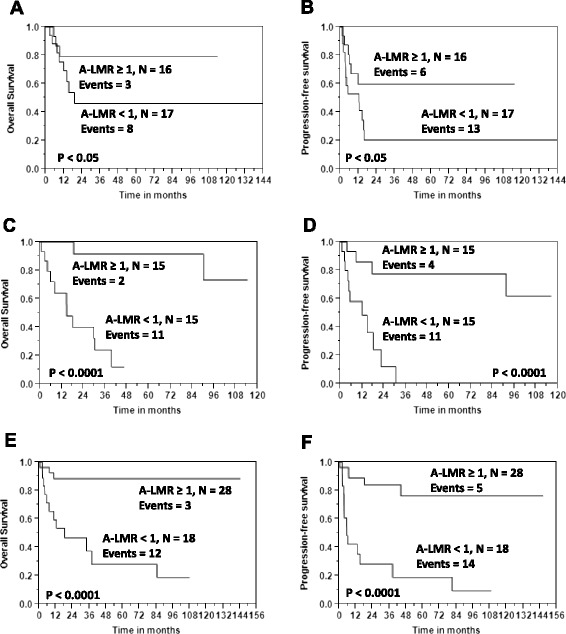
Overall survival: **a** peripheral T-cell lymphoma, **c** angioimmunoblastic T-cell lymphoma, and **e** others. Progression-free survival: **b** peripheral T-cell lymphoma, **d** angioimmunoblastic T-cell lymphoma, and **f** others

Using the cut-off value of 1.0 for the A-LMR obtained from the empiric ROC and subsequently validated by *k*-fold cross-validation, we tested A-LMR ≥ 1 for OS and PFS. We observed that patients infused with an A-LMR ≥ 1 compared with patients infused with an A-LMR < 1 experienced superior OS (Fig. 4a) and PFS (Fig. 4b) [median OS was not reached versus 17.9 months, 5-year OS rates of 87 % (95 % CI, 75–94 %) versus 26 % (95 % CI, 13 %-42 %), p < 0.0001; median PFS was not reached versus 11.9 months, 5-year PFS rates of 72 % (95 % CI, 58–83 %) versus 16 % (95 % CI, 6–32 %), *p* < 0.0001]. By histopathological subtype, superior OS and PFS was observed in patients with an A-LMR ≥ 1 compared with patients with an A-LMR < 1 diagnosed with peripheral T-cell lymphoma not otherwise specified (NOS) [median OS (Fig. 5a) was not reached versus 18.7 months, 3-year OS rates of 79 % (95 % CI, 70–93 %) versus 46 % (95 % CI, 23–71 %), *p* < 0.05; median PFS (Fig. 5b) was not reached versus 12.2 months, 3-year PFS rates of 60 % (95 % CI, 51–91 %) versus 21 % (95 % CI, 7–47 %), *p* < 0.0001], for angioimmunoblastic T-cell lymphoma [median OS (Fig. 5c) was not reached versus 14.6 months, 3-year OS rates of 92 % (95 % CI, 59–99 %) versus 24 % (95 % CI, 8–54 %), *p* < 0.0001; median PFS (Fig. 5d) was not reached versus 11.9 months, 3-year PFS rates of 78 % (95 % CI, 48–93 %) versus 0 %, *p* < 0.0001], and for others [median OS (Fig. 5e) was not reached versus 18.1 months, 3-year OS rates of 88 % (95 % CI, 70–96 %) versus 28 % (95 % CI, 10–57 %), *p* < 0.05; median PFS (Fig. 5f) was not reached versus 5.3 months, 3-year PFS rates of 74 % (95 % CI, 64–94 %) versus 28 % (95 % CI, 12–54 %), *p* < 0.0001].

## Discussion

In B-cell lymphoma, the A-LMR has been reported to be a prognostic factor for survival in patients treated with APHSCT. Thus, we set out to investigate if the A-LMR can impact survival in patients with T-cell lymphomas treated with APHSCT.

In all T-cell lymphoma patients undergoing APHSCT, the infusion of an A-LMR ≥ 1 was associated with superior OS and PFS. Furthermore, A-LMR showed a homogeneous prognostic role between the histological subtypes, as superior survival was observed in patients with an infused A-LMR ≥ 1 regardless of the underlying T-cell lymphoma histologies. This observation was observed in subsets of patients categorized by histological subtypes as well. Multivariate analysis revealed the A-LMR to be an independent prognostic factor for survival in T-cell lymphoma patients treated with APHSCT when adjusted for other reported prognostic factors.

The ALC-15 has been reported to be a prognostic factor for survival in B-cell lymphomas treated with APHSCT and has also been reported to be a prognostic factor for survival in T-cell lymphoma patients undergoing APHSCT. The ALC-15 recovery is directly dependent on the amount of collected and infused A-ALC. In this study, as we published in B-cell lymphomas [12], the correlation between A-ALC and ALC-15 was confirmed for the first time in T-cell lymphomas treated with APHSCT. *In vitro* and clinical studies have reported inhibition of host immunity and survival by immunosuppressive monocytes in T-cell lymphoma [18]. In APHSCT, we documented that A-AMC, which directly affects AMC-15 recovery, conveyed a negative prognosis in B-cell lymphomas. We observed the same negative survival effect in T-cell lymphoma patients infused with higher A-AMC and AMC-15 numbers post-APHSCT. As in our previous reports [12, 13, 15], no correlation was identified between the infused CD34 count and ALC-15, AMC-15, or LMR-15 in T-cell lymphoma patients undergoing APHSCT. To our knowledge, this is the first study reporting that the A-LMR, which combined the biomarkers of A-ALC and A-AMC, not only affects ALC-15 and AMC-15 recovery but also survival in T-cell lymphoma patients treated with APHSCT. The possible mechanism addressing the negative survival of patients infused with an A-LMR < 1 due to the higher content of A-AMC has been addressed in our previous publication [19].

The limitations of our study include being a retrospective study and a small cohort of T-cell lymphomas undergoing APHSCT. The strengths of the study include long-term follow-up of T-cell lymphoma patients treated consecutively with APHSCT at a single institution. This study expands on the previous publications regarding A-ALC and A-AMC by highlighting the importance of the interaction between host immunity and tumor microenvironment, using the simple biomarkers of A-ALC and A-AMC combined in the prognostic factor of A-LMR.

## Conclusion

Finally, the association between A-LMR and survival provides a rationale to develop clinical translational interventions to engineer immunocompetent autografts with direct impact on immune recovery and survival, not only in patients with B-cell lymphomas but also T-cell lymphomas treated with APHSCT.

## Materials and methods

### Patient population

To qualify for the study, patients were required to be candidates for APHSCT with the diagnosis of T-cell lymphoma and have mobilized enough peripheral blood stem cells to proceed with APHSCT (minimum of 2.0 × 10^6^ CD34 cells/kg). Patients were excluded if they failed to mobilize stem cells, required bone marrow harvest, were infused with both peripheral blood and bone-marrow-harvest-derived stem cells, or participated in stem cell transplantation clinical trials. No patients were lost to follow-up. From 1998 to 2014, 109 T-cell lymphoma patients qualified for the study. All patients gave written, informed consent allowing the use of their medical records for medical research. Approval for the retrospective review of these records was obtained from the Mayo Clinic Institutional Review Board and was in accordance with US federal regulations and the Declaration of Helsinki.

### End points

The primary end point of the study was to assess the impact of A-LMR on overall survival (OS) and progression-free survival (PFS) from the time of APHSCT in patients with T-cell lymphoma. The infused A-ALC for each apheresed unit collection was calculated as follows: A-ALC = % collection lymphocytes × (absolute white blood cell (WBC) count/kg). The infused A-AMC for each apheresed unit collection was calculated as follows: A-AMC = % collection monocytes × (absolute WBC count/kg). The A-LMR was then calculated by dividing the A-ALC by the A-AMC.

### Prognostic factors

The following prognostic factors were evaluated in the study: international prognostic score (IPI) [20] at diagnosis: [age (>60 years), extra-nodal disease (>1 site), lactate dehydrogenase (LDH), performance status (>1), and stage]; A-LMR; A-ALC; A-AMC; ALC-15; AMC-15; day 15 lymphocyte-to monocyte ratio (LMR-15); B symptoms; bone marrow involvement at diagnosis; bulky disease (>10 cm); clinical status prior to APHSCT [complete response (CR) versus partial response (PR)]; hemoglobin (<12 g/dl) at diagnosis; platelets (<150 × 10^9^/l) at diagnosis; and infused CD34+ stem cells dose.

### Peripheral blood stem cell (autograft) collections

Patients received granulocyte colony-stimulating factor (G-CSF) for mobilization at a dose of 10 μg/kg daily for 5–7 consecutive days by subcutaneous injection. Once the peripheral blood CD34+ cell count was ≥10 cells/μl on G-CSF, patients began daily apheresis until a minimum target of 2.0 × 10^6^ CD34 cells/kg was reached. If on day 4 on G-CSF, the peripheral blood CD34 was less than 10 cells/μl, the addition of plerixafor 0.24 mg/kg was allowed.

### Conditioning regimen

All patients were treated with BEAM: BCNU (300 mg/m^2^) on day −6, etoposide (100 mg/m^2^) twice daily from days −5 to −2, cytarabine (100 mg/m^2^) twice daily from days −5 to −2, and melphalan (140 mg/m^2^) on day −1.

### Response and survival

Response criteria were based on the guidelines from the International Harmonization Project on Lymphoma [21]. OS was measured from the date of transplant to the date of death or last follow-up. PFS was defined as the time from transplant to the time of progression, relapse, death, or last follow-up.

### Statistical analysis

OS and PFS were analyzed using the approach of Kaplan and Meier [22]. Differences between survival curves were tested for statistical significance using the two-tailed log-rank test. The Cox proportional hazard model [23] was used for the univariate and multivariate analyses to evaluate the variables under the “Prognostic factors” section to assess their impact on post-APSCHT OS and PFS times. The choice of optimal cut-off for A-LMR, A-ALC, A-AMC, ALC-15, AMC-15, and LMR-15 to assess survival was based on their utility as a marker for the clinically relevant binary outcome of death/survival using the receiver operating characteristic (ROC) curves and area under the curve (AUC). The binary clinical outcome (death/survival) was established at 5 years post-APHSCT. Patients were classified as “alive/censored” when follow-up time was greater than 5 years and “death” for patients known to have died before this time point [24]. A *k*-fold cross-validation with *k* values of 10 was performed to validate the results of A-LMR, A-ALC, A-AMC, ALC-15, AMC-15, and LMR-15. Based on this analysis, cross-validation AUC by the ROC was produced, representing the discriminating accuracy of A-LMR, A-ALC, A-AMC, ALC-15, AMC-15, and LMR-15 for the binary clinical outcomes of death/survival. To assess the reproducibility of the cut-off value for A-LMR obtained by the ROC curves, the relationship between A-LMR as a continuous variable and the logarithm of the hazard ratio was explored using the Cox proportional hazard restricted cubic spline regression in regard to OS and PFS. The cut-off value was chosen at the point where the A-LMR value caused the hazard ratio to change from favorable to unfavorable.

*Χ*^2^ tests and Fisher exact tests were used to determine relationships between categorical variables as appropriate. The Wilcoxon rank test was used to determine associations between continuous variables and categories, and nonparametric tests were used to evaluate associations for continuous variables. All *p* values represented were two-sided, and statistical significance was declared at *p* < 0.05.

## Abbreviations

A-ALC: autograft absolute lymphocyte count
A-AMC: autograft absolute monocyte count
ALC-15: day 15 absolute lymphocyte count post-autologous peripheral hematopoietic stem cell transplantation
A-LMR: autograft lymphocyte/monocyte ratio
AMC-15: day 15 absolute monocyte count post-autologous peripheral hematopoietic stem cell transplantation
APHSCT: autologous peripheral hematopoietic stem cell transplantation
AUC: area under the curve
BEAM: BCNU, etoposide, cytarabine, and melphalan
CI: confidence interval
CR: complete response
IPI: International Prognostic Index
LDH: lactate dehydrogenase
LMR-15: day 15 lymphocyte/monocyte ratio post-autologous peripheral hematopoietic stem cell transplantation
OS: overall survival
PFS: progression-free survival
PR: partial response
ROC: receiver operating characteristic
